# Supplementary material for: Risk factors for sacrococcygeal pilonidal sinus: a systematic review and meta-analysis supplemented by genetic causal assessment
Source: Front Surg. 2026 Jan 7;12:1718589. doi: 10.3389/fsurg.2025.1718589 (PMC12819706; doi:10.3389/fsurg.2025.1718589)
Supplement: Supplementary file 2 [file Datasheet2.zip › Supplementary Data 2/MR_pipeline_p5e-8/leaveone_plots/ukb-b-223.finngen_R12_L12_PILONIDALCYST.pdf]

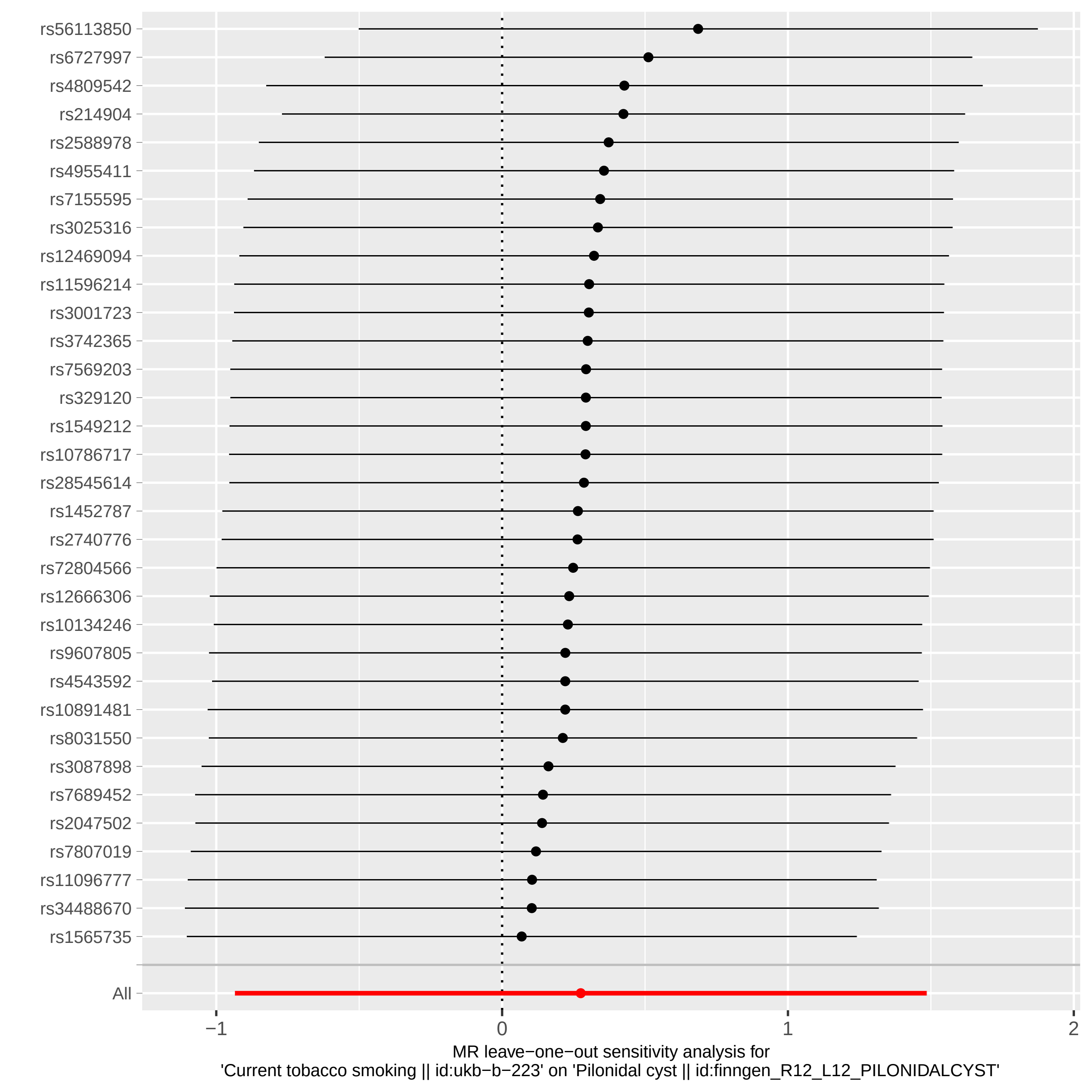
rs56113850
rs6727997
rs4809542
rs214904
rs2588978
rs4955411
rs7155595
rs3025316
rs12469094
rs11596214
rs3001723
rs3742365
rs7569203
rs329120
rs1549212
rs10786717
rs28545614
rs1452787
rs2740776
rs72804566
rs12666306
rs10134246
rs9607805
rs4543592
rs10891481
rs8031550
rs3087898
rs7689452
rs2047502
rs7807019
rs11096777
rs34488670
rs1565735
All
−1
0
1
2
MR leave−one−out sensitivity analysis for
'Current tobacco smoking || id:ukb−b−223' on 'Pilonidal cyst || id:finngen_R12_L12_PILONIDALCYST'
